# Glutamate as a Stressoric Factor for the Ex Vivo Release of Catecholamines from the Rabbit Medial Prefrontal Cortex (mPFC)

**DOI:** 10.3390/life11121386

**Published:** 2021-12-11

**Authors:** Bogdan Feliks Kania, Danuta Wrońska, Izabela Szpręgiel, Urszula Bracha

**Affiliations:** 1 Veterinary Institute, University Center for Veterinary Medicine Jagiellonian University & Agriculture University, Hugon Kollataj Agricultural University in Cracow, 30-059 Krakow, Poland; 2 Department of Physiology and Endocrinology of Animals, Faculty of Animal Sciences, Hugon Kollataj Agricultural University in Cracow, 30-059 Krakow, Poland; danuta.wronska@urk.edu.pl (D.W.); izabela.szpregiel@student.urk.edu.pl (I.S.); 3 Center of Experimental and Innovative Medicine, Hugon Kollataj Agricultural University in Cracow, 30-248 Krakow, Poland; urszula.bracha@urk.edu.pl

**Keywords:** medial prefrontal cortex, glutamate, release of catecholamines, rabbit

## Abstract

One of the major roles of glutamic acid (Glu) is to serve as an excitatory neurotransmitter within the central nervous system (CNS). This amino acid influences the activity of several brain areas, including the thalamus, brainstem, spinal cord, basal ganglia, and pons. Catecholamines (CAs) are synthesized in the brain and adrenal medulla and by some sympathetic nerve fibers. CAs, including dopamine (DA), norepinephrine (NE), and epinephrine (E), are the principal neurotransmitters that mediate a variety of CNS functions, such as motor control, cognition, emotion, memory processing, pain, stress, and endocrine modulation. This study aims to investigate the effects of the application of various Glu concentrates (5, 50, and 200 µM) on CAs release from rabbit medial prefrontal cortex (mPFC) slices and compare any resulting correlations with CAs released from the hypothalamus during 90 min of incubation. Medial prefrontal cortex samples were dissected from decapitated, twelve-week-old female rabbits. The results demonstrated that Glu differentially influences the direct release of CAs from the mPFC and the indirect release of CAs from the hypothalamus. When under stress, the hypothalamus, a central brain structure of the HPA axis, induces and adapts such processes. Generally, there was an inhibitory effect of Glu on CAs release from mPFC slices. Our findings show that the effect arises from Glu’s action on higher-order motivational structures, which may indicate its contribution to the stress response by modulating the amount of CAs released.

## 1. Introduction

The medial prefrontal cortex (cortex preafrontalis medialis, mPFC) is the part of the frontal lobe of the cerebral cortex that is located frontmost and covers the orbital region. When we consider evolution, it is the youngest region of the brain. The neurons of this cortex are characterised by long dendrites covered with a large number of dendritic spines—projections receiving stimulus inputs from other neurons through the synapse at its top. The dendrite of one neuron can be covered with thousands of dendrite spines that perfectly increase the ability to make contact with a large number of other neurons.

The prefrontal cortex controls the operating memory, plans movements and actions, and analyses their consequences. Playing a crucial role in higher cognitive processes, it also inhibits spontaneous and often violent and detrimental emotional states arising in the hypothalamus and the limbic system [1]. The prefrontal cortex mainly controls behavior and adapts it to environmental conditions. These roles are attributed, in particular, to the mPFC [2,3], which assesses and interprets information, and then—using previous experience—generates behavior appropriate to the current social situation [4].

The medial part of the mPFC sends projections to many important brain regions involved in social behavior: the amygdala (cerebral emotion centre), accumbens nucleus (cerebral pleasure centre), hippocampus (memory centre), and brainstem [2].

Prefrontal lobotomies (cutting off the mPFC from the rest of the brain), conducted in the 1940s and 1950s on monkeys, demonstrated that the animals became calm but also did not show changes in sensory or motor (movement) functions. However, in most of the animals tested, prefrontal lobotomies caused negative effects in the form of apathy, inability to plan and take the initiative, memory impairment, susceptibility to distraction, and the loss of ability to express emotions [1].

The prefrontal cortex is responsible for inhibiting or stimulating other parts of the brain, depending on whether the decision being made is to generate some behavior or refrain from it. The prefrontal cortex’s proper functioning is the result of appropriate recognition of the stressor, which ensures that excitation—caused by stress—is maintained at the proper level. The prefrontal cortex also guarantees the adequacy of stress responses.

Primary projections of glutamatergic signalling—according to some to the mPFC and according to others to the tegmentum area—are considered the primary signalling sites for the glutamatergic cortical DA regulation of stress [5].

The mPFC covers a large area of the frontal lobe and creates programs of complex actions implemented by areas of the sensory–motor cortex. In humans, the mPFC is very important for intellectual phenomena, abstract thinking, and planning and completing complicated activities [6].

The mPFC has many pathways with other cortical areas and subcortical structures, which are organised in a topographic manner so that the structures that regulate emotions are located ventrally and medially (hypothalamus and amygdala) [7]. These are affected by DA and NE. Under stress, DA affects the hypothalamus and amygdala, and NE affects the hypothalamus and cerebral cortex. The amygdala affects the DA of the hypothalamus and striatum and causes the abolition of regulation by the mPFC.

The dorsolateral mPFC has numerous connections with the sensory and motor cortex and is crucial for regulating attention, thinking, and acting.

The ventromedial prefrontal cortex (vmPFC) has numerous connections with subcortical structures such as the amygdala, nucleus accumbens, and hypothalamus, which create strength responses and habits, and are, therefore, able to regulate emotional reactions [7].

Ultimately, the dorsomedial prefrontal cortex (dmPFC) is associated with error monitoring in human MRI functional and diagnostic tests. The PFC regions cooperate in regulating higher-order decisions, including planning and organising for the future.

Under stress-free conditions, the connections of the mPFC organise brain activity for the intelligent regulation of behavior, thinking, and emotions.

The mPFC also has direct and indirect relations with the monoaminergic cells of the brainstem, such as the locus coeruleus (where large NE-projections reach) and the substantia nigra and VTA (where large DA-ergic projections stretch), and, thus, is able to regulate its CA-ergic conductivity. In turn, when optimal amounts of CA are released, they intensify the regulation of the mPFC, creating an “excellent cycle” [8].

During psychological stress, the amygdala stimulates the hypothalamus and brainstem stress pathways, which release high levels of NE and DA. Thus, under stress conditions, the image switches from a slow reaction and cortical regulation to violent emotional reactions of the amygdala and the respective subcortical structures [9].

Moderate, uncontrolled stress can cause a rapid and dramatic loss of prefrontal cortical cognition, and stress that is more chronic can cause changes in the structure of the prefrontal cortex. Intracellular signalling pathways are involved in the effects of mPFC stress [9,10].

Projections into the mPFC, e.g., in rabbits, originate from the base of the midbrain, lateral hypothalamus, septal nuclei, locus coeruleus, and dorsomedial and other thalamic nuclei. The efferent connections of the mPFC were described by Buchanan et al. (1994) [8]. Areas 24, 25, and 32 have different efferent connections with other cortical and subcortical areas (striatum, caudate nucleus, and mesocortex). The projection of area 25 reaches the thalamus, lateral hypothalamus, amygdala, PAG, septum nucleus, VTA, substantia nigra, locus coeruleus, pons nuclei, colliculi of corpora quadrigemina, and dorsal and ventral cortex. Together, these connections play a role in associative learning [8,10].

Stress increases Glu concentration in the mPFC; however, the mechanisms of this phenomenon are not yet fully understood. Lupinsky et al. (2010) [11], using microdialysis and the local application of agents, examined the post-stress relationships in Glu/mPFC reactions in rats, which may reflect increased inter-septal communication through the projection neurons of the corpus callosum. Lupinsky et al. found that a 20 min tail-flick test resulted in a comparable increase in Glu release in both the left and right mPFCs. This release was dependent on the presence of Na^+^ and Ca^2+^ ions and was not affected by cysteine, which, under normal conditions, blocks the release of Glu. Unilateral mPFC lesions induced by the application of ibotenate abolished the post-stress Glu release reactions in the mPFC of the hemisphere opposite to the damaged one, as a result of stimulation of mGluR_2/3_ receptors in the opposite hemisphere. The topical application of the DA_1_ receptor blocker in the left mPFCs potentiated an increase in the concentration of Glu released as a stress response in the right mPFCs. The same treatment for the right mPFCs had far less effect on the release of Glu from the cortex in reaction to stress. Therefore, the release of Glu by the mPFC as a stress response was abolished or potentiated by blocking α_1_-adrenergic receptors and stimulating GABA_B_ receptors, respectively, in opposing hemispheres. The results indicate that the reactions increasing the release of Glu in the mPFC by stress at least partially reflect the activation of neurons located in the corpus callosum of the opposite hemispheres. They also prove that the induced activation of neurons by a stress factor is regulated by Glu, DA, NE, and GABA-sensitive mechanisms. In the case of DA, this control is asymmetrical, with a significant regulatory effect of the left mPFC, because its DA response is stronger—as a response to the stressor in the form of Glu release—than that of Glu in the right mPFC. These data suggest that the corpus callosum neurons and their afferentation play a very important role in hemispherical mPFC specialization mediating stress responses [12]. Abnormally increased monoamine (DA) transmission in the mPFC is secondary to anomalies in glutamatergic cortical neurotransmission. Understanding these processes is fundamental for the pathophysiology of psychiatric disorders that are a consequence of stress and will help in planning new strategies for both the prevention and treatment of these diseases. The stimulation of Glu transmission in the mPFC under stress conditions is a common mechanism through which stress affects normal and abnormal processes that abolish or maintain affection and cognition [12].

This study aimed to determine the effect of various L-Glu concentrations—the primary excitatory amino acid/transmitter in the CNS—on CA release from mPFC slices. This brain structure has high concentrations of both glutamatergic and catecholaminergic receptors. Glu concentrations exceeding 5, 50, and 200 times its physiological concentration were used. Excess Glu in brain neurons causes a strong depolarization of neural presynaptic terminals. Excessive Glu can cause neurotoxic or neurodegenerative effects leading to degenerative changes and intensifying the release of various transmitters, including CAs, in the adrenergic pathways of the brain in vivo. The ex vivo method made it possible to exclude any other effects of the intracerebral neural systems on the changes in CA concentrations obtained after applying different concentrations of Glu as a stress factor. The species, rabbit, selected for the experiments is particularly sensitive to changes in environmental conditions, especially those considered stress factors.

## 2. Material and Methods

Just after the heart and breathing stopped, the naive rabbits were decapitated. The cranial skin was incised by cranium trepanning. The brain was removed and put on ice. The mPFC tissues were extracted [13,14]. The mPFCs were obtained from twelve decapitated twelve-week-old female rabbits at the Experimental Station of the Department of Animal Biotechnology of the Agricultural University in Krakow-Bielany. All experimental procedures were approved by the Local Bioethics Committee at the Jagiellonian University in Krakow (approval No 75/2007). After decapitation, the brains were collected and placed in 0.9% NaCl, and the brain structures were dissected. The pieces of mPFC tissue (about 50 mg) were isolated from the white matter of the prefrontal cortex (according to the Atlas of the rabbit brain and spinal cord: Shek et al., 1986) and then cut with scissors into tiny slices that were placed in incubation wells (cell culture SIGMA) containing 1 mL of Eagle’s incubation medium (Krebs phosphate buffer containing 0.3% glucose and 0.1% BSA) without (control group) or with (study group) three doses of L-Glu (Sigma Aldrich, St. Louis, MO, USA) in concentrations of I-5, II-20, or III-50 µM in a volume of 10 μL. Every 30 min, each slice of the mPFC tissue was placed in the next well with the medium. The incubation was done in a carbogen atmosphere of 95% O_2_ and 5% CO_2_ at 38 °C in a Sanyo incubator (MCO-18AIC). The medium collected after 30, 60, and 90 min of the experiment was used for DA, NE, and E radioimmunoassay (RIA) measurement. A rapid staining procedure using fluorescein diacetate was utilized to detect cell viability in cell suspension [15,16].

The radioactivity of the samples was measured in a gamma counter “Wizard” (LKB, Vienna, Austria). The sensitivity of E was 19 pg·mL^−1^, the intra-run error was 10.1%, and the inter-run error was 9.2%, respectively. For NE, it was 0.2 pg·mL^−1^, with an intra-run error equal to 12.3% and an inter-run error of 10.9%, respectively. For DA, it was 0.10 pg·mL^−1,^ with an intra-run error equal to 12.3% and an inter-run error of 22.7%, respectively.

### Statistical Treatment of Results

The results were analysed statistically using a two-way analysis of variance for repeated measurements. The significance of differences between mean values was determined by Duncan’s test. Calculations were performed using Statistica v.13.1 software (StatSoft, Inc., Tulsa, OK, USA). A probability of *p* < 0.05 indicated statistically significant differences between the mean values.

## 3. Results

**Figure 1:**
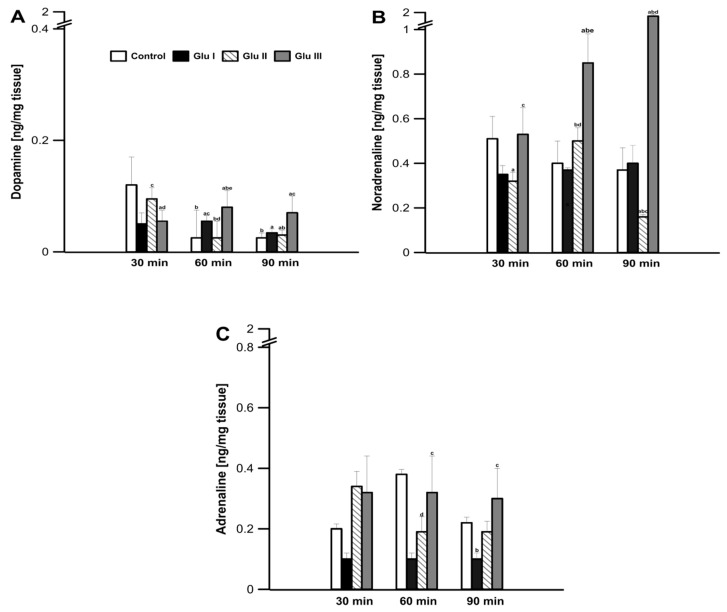
Changes in the concentration of dopamine (**A**), noradrenaline (**B**), and adrenaline (**C**) released from rabbit mPFC tissue slices after 30, 60, and 90 min of incubation in the presence of added glutamate (L-Glu) at a concentration of 5 μM (Glu I), 50 μM (Glu II), and 200 μM (Glu III). Values are means ± SEM (*n* = 12). Values marked with different letters differ significantly at *p* < 0.05.

During the control incubation of prefrontal cortex tissue in the incubation medium, dopamine concentration in the tissue was determined to be 0.12 ± 0.02 ng·mg^−1^ after the first 30 min of the experiment (Figure 1A). After another 30 min, the concentration of dopamine decreased to a value of 0.012 ± 0.001 ng·mg^−1^ of mPFC tissue (*p* < 0.05) and remained at this level until 90 min into the experiment. The use of Glu I resulted in a significant reduction in the concentration of dopamine after the first 30 min of the experiment to the value of 0.05 ± 0.006 ng·mg^−1^ of mPFC tissue (*p* < 0.05), compared to the values found in the control group, after which it did not change significantly in the minutes following, reaching a value of 0.04 ± 0.002 ng·mg^−1^ of mPFC tissue (*p* < 0.05) at 90 min. The higher dose of Glu II used to incubate the strips of mPFC did not reduce the concentration of dopamine after the first 30 min of the experiment (0.10 ± 0.007 ng·mg^−1^ of tissue; *p* > 0.05). After the next 30 min, the concentration of dopamine decreased to the value of 0.01 ± 0.008 ng·mg^−1^ of tissue (*p* > 0.05), and a value of 0.03 ± 0.03 ng·mg^−1^ of mPFC tissue was determined after 90 min (Figure 1A). The highest dose of Glu used to incubate the mPFC tissues caused a decrease in dopamine concentration after the first 30 min of the experiment (0.05 ± 0.005 ng·mg^−1^ of tissue; *p* < 0.05). After another 30 min, an increase in the concentration of dopamine in the incubation medium was observed, and the value found (0.08 ± 0.009 ng·mg^−1^ of tissue; *p* > 0.05) turned out to be significantly greater than that found at the same time in the control and other two experimental groups (*p* > 0.05). In the last measurement, after 90 min of the experiment, a value of 0.07 ± 0.01 ng·mg^−1^ of tissue was found in this group, which turned out to be significantly higher than all other groups (*p* > 0.05; Figure 1A).

During the experiment, in the prefrontal cortex tissue, no significant changes in the amount of noradrenaline excreted into the medium were found in the control group (values ranging from 0.51 ± 0.10 to 0.53 ± 0.09 ng·mg^−1^ of tissue; *p* > 0.05; Figure 1B). The use of Glu I and Glu II for the incubation of the investigated tissue resulted in a significant decrease in the concentration of catecholamine in the medium (0.31 ± 0.03 and 0.28 ± 0.04 ng·mg^−1^ of tissue; *p* < 0.05, respectively); Glu III showed no significant changes compared to the values found in the control group. As the experiment continued, the concentration of noradrenaline in the medium increased significantly after the highest dose of Glu III was applied, up to the value of 1.59 ± 0.25 ng·mg^−1^ of tissue found after 90 min of the experiment (*p* < 0.05; Figure 1B).

After the first 30 min of the experiment, no significant changes in the amount secreted to medium A from the prefrontal cortex tissue were observed, and the values found in all groups did not differ significantly (*p* > 0.05; Figure 1C). In the following minutes of the experiment, only the Glu II group showed a decrease in the amount excreted into medium A, while in the control group and the other two experimental groups, the amount excreted into medium A did not change (Figure 1C).

## 4. Discussion

According to some authors, the reported Glu concentrations in the rabbit mPFCs are around 22 µM [16], while in rats, Glu concentration is 2.216·10^−6^ M, DA-1.81·10^−10,^ and NE 2.786·10^−10^ M [14]. The concentration of DA increases in the mPFC of conditioned rats compared to control animals. Similarly, the NE concentration was significantly elevated in the mPFCs of conditioned rats subjected to stress relief training. In the mPFC of normothermic rats, the Glu concentration in the dialysate ranged from 12 to 16 µM and from 25 to 28 µM during hypothermia [17].

The results show the inhibitory effect of the physiological concentration of Glu (5 µM) [17] on the release of DA, NE, and E after a 30 min incubation of the mPFC slices and the enhancing effect of the remaining 10 and 40 times higher concentrations than physiological Glu levels (50 and 200 µM) on the release of DA and NE. Similar results, indicating a statistically significant inhibitory effect on CA release for all three Glu concentrations used in the study, were found in the hypothalamus slices [18] and rabbit amygdala [16] during 90 min of observation. The different effects of high concentrations of Glu, increasing the DA and NE release from rabbit mPFC slices by as much as 200–700%, were found after 60 and 90 min of incubation. This result is difficult to interpret. High concentrations of Glu (50 and 200 µM) 60 min after the start of the incubation of rabbit mPFC slices presumably resulted in either the increase of CA release by destroying the neurons contained in the homogenates or the intensification of interactions found in slices of glial cells stimulating neurons to release the pools of free CA stored in them, which was not analysed in the study.

However, the neurons of motivational structures, including mPFCs, contain both ionotropic glutamatergic receptors (iGluR-NMDA, AMPA, and kainate) and metabotropic receptors (mGluR-groups I, II, and III), as well as catecholaminergic receptors (CA-ergic), especially noradrenergic receptors (NE-ergic) and dopaminergic (DA-ergic) [19]. CA-ergic receptors are most often autoreceptors.

Glu is the most potent excitatory neurotransmitter in humans, animals, and plants [20]. It is present in the CNS, AUN, and peripheral organs of humans and animals. When released from neurons, Glu stimulates both iGluR and mGluR receptors, located presynaptically (groups II and III) and postsynaptically (group I mGluR). The excitation of postsynaptic receptors causes their depolarization and intensification of transmission in various neuronal systems (e.g., DA, NE, E, 5-HT, and GABA). The stimulation of glutamatergic autoreceptors inhibits the release of Glu from glutamatergic neurons and, thus, reduces its excitatory effect on a number of other neural systems of a different nature. The influence of Glu on catecholamine metabolism in the CNS, especially in ex vivo mPFC slices in rabbits, is more complicated. The mPFC slice comprises a mixture of neurons and glial cells containing more than just glutamatergic receptors, which were affected by added Glu. As mentioned earlier, apart from Glu-ergic receptors, DA-ergic receptors, especially NE-ergic ones, and autoreceptors are present on mPFC neurons. By depolarizing its autoreceptors, Glu reduces its release from neurons; however, it also depolarizes the neighboring DA- and NE-ergic receptors with a similar outcome, which would result in its statistically significant effect of increasing CA release from rabbit mPFC slices. Fukuyama et al. (2020) [21] claim that in the Glu-ergic pyramidal neurons of the rat frontal cortex, postsynaptic alpha_2_-adrenergic receptors are present. Thus, the intensification of Glu-ergic stimulation would result in the simultaneous release of CA and stimulation of CA-ergic receptors, which, in turn, would lead to the inhibition of the activity of cortical neurons. At this stage of ex vivo research, it would be difficult to assume, as the authors cited above, that the same happens in vivo. Using the multi-probe microdialysis method with ultra-high performance liquid chromatography, they determined that stimulation of the alpha_2_ receptor in the locus coeruleus inhibited NE release in the frontal cortex did not affect DA release. Chronic stimulation of this alpha_2_ receptor did not affect GABA-ergic transmission; however, it phasically enhanced thalamocortical Glu-ergic transmission [21].

The results obtained after using high doses of Glu, consisting of a statistically significant increase in DA and NE release from rabbit mPFC sections, proved that Glu at the concentrations used should be considered a stress factor for mPFC tissues, which is further supported because in vivo, the release of Glu from the rat mPFC increases under stress. Thus, for the first time in a rabbit in vitro, a simple correlation was found between an increase in Glu concentration and a simultaneous increase in CAs release from the same brain structure. In other words, the increased concentration of Glu in the medium of the rabbit mPFC sections was an alarm response for the mPFC neurons in the incubation fluid. Perhaps the earlier use of alpha_2_-adrenoceptor inhibitors could reduce or inhibit the post-stressoric release of CAs, since NMDARs antagonists did not, the agonist of which -is Glu, apart from aspartic acid. However, these results were obtained in studies on the effect of Glu on the release of radiolabelled NE from rat PFC sections [22].

In the future, it would be advisable to investigate the influence of glutamic acid on direct and indirect CAs release in both male and female rabbits since numerous animal studies demonstrate differences in susceptibility to stress between the sexes, with males usually being more vulnerable [23,24]. Moreover, some studies indicate that gamma-Aminobutyric acid (GABA) is also involved in the release of catecholamines [25,26]; it would be interesting to compare these two neurotransmitters in terms of their influence on the regulation of CAs. Further research into factors impacting the release of catecholamines is unquestionably needed, especially since disturbances in CA levels are linked to various clinical diseases, such as Parkinson’s disease, Alzheimer’s disease, depression, and anxiety [27,28,29,30,31]. Understanding the mechanisms behind the regulation of the release of catecholamines is crucial for further research into the aforementioned disorders.
